# Prevalence and Clinical Correlation of Intron 22 Inversion in Hemophilia A in Northeast India

**DOI:** 10.7759/cureus.82365

**Published:** 2025-04-16

**Authors:** Ruby Barman, Adity Sarma, Zarika Ahmed, Anupam Dutta

**Affiliations:** 1 Department of Pathology, Assam Medical College and Hospital, Dibrugarh, IND; 2 Department of General Medicine, Assam Medical College and Hospital, Dibrugarh, IND

**Keywords:** factor viii deficiency, genetic mutation analysis, hemophilia a, intron 22 inversion, molecular diagnostics

## Abstract

Background: Hemophilia A is an X-linked bleeding disorder caused by mutations in the F8 gene, with intron 22 inversion being the most common genetic alteration in severe cases. This study aimed to determine the prevalence of intron 22 inversion in hemophilia A patients in Assam, Northeast India, and evaluate its correlation with disease severity.

Methods: This hospital-based observational study included 80 hemophilia A patients at Assam Medical College from March 2023 to February 2024. Clinical history, coagulation tests (activated partial thromboplastin time (APTT), prothrombin time (PT), and factor VIII assay), and nested long-distance polymerase chain reaction (NLD-PCR) for intron 22 inversion detection were performed. Statistical analysis was conducted using SPSS v25 (IBM Corp., Armonk, New York, USA).

Results: Among the 80 patients, 41 (51.25%) had severe hemophilia A, 22 (27.5%) moderate, and 17 (21.25%) mild. Intron 22 inversion was detected in 24 patients (30%), with a significantly higher prevalence in severe cases 22/41 (53.65%) compared to moderate 2/22 (9.09%) and mild cases (0%) (p < 0.001). APTT was significantly prolonged in severe cases (109.35 ± 15.62 sec) compared to moderate (86.92 ± 9.87 sec) and mild cases (61.71 ± 8.94 sec) (p < 0.001), reinforcing the genetic basis of disease severity.

Conclusion: This study confirms a strong correlation between intron 22 inversion and severe hemophilia A, consistent with global and Indian studies. It emphasizes the need for routine genetic screening for early diagnosis and personalized treatment strategies, including genetic counseling. Future research should explore other F8 mutations, gene therapy, and the role of intron 22 inversion in inhibitor development.

## Introduction

Hemophilia A is a hereditary bleeding disorder caused by a deficiency or dysfunction of clotting factor VIII (FVIII), a crucial protein in the intrinsic pathway of the coagulation cascade. It is inherited in an X-linked recessive manner, meaning that it primarily affects males, while females typically act as asymptomatic carriers. The global incidence of hemophilia A is estimated to be around one in 5,000 to 10,000 live male births [1,2]. This condition is characterized by prolonged bleeding, which can occur spontaneously or after minor trauma, and commonly manifests as bleeding into joints (hemarthrosis), muscles, or internal organs, potentially resulting in joint damage, chronic pain, and disability if not adequately managed [3,4].

The severity of hemophilia A correlates with the residual activity of FVIII in plasma. Severe hemophilia A is defined by FVIII activity of less than 1% of normal and is often associated with frequent spontaneous bleeding episodes [5]. Genetic mutations in the F8 gene, which encodes FVIII, underlie this disorder. Among the various mutations identified, the intron 22 inversion (Inv22) is the most common cause of severe hemophilia A, accounting for approximately 40% to 50% of cases worldwide [6-8].

The Inv22 mutation is a large structural rearrangement caused by intrachromosomal homologous recombination between the int22h-1 region within intron 22 of the F8 gene and one of two extragenic copies (int22h-2 or int22h-3) located approximately 0.5 Mb telomeric to the gene on the X chromosome [9]. This recombination event inverts a large segment of the gene, disrupting the coding sequence and preventing the proper production of the FVIII protein. As a result, affected individuals produce either a truncated or nonfunctional FVIII protein, leading to severe disease [10]. This inversion does not delete any portion of the gene but renders it transcriptionally inactive due to incorrect orientation of the exons and promoters [11].

Detection of the Inv22 mutation is critical not only for confirming a diagnosis of hemophilia A but also for genetic counseling, prenatal diagnosis, and carrier detection in families at risk. Importantly, several studies have also reported that patients with Inv22-positive hemophilia A are at a higher risk of developing inhibitors, which are neutralizing antibodies against administered FVIII [12,13]. The development of inhibitors complicates disease management, as it reduces the effectiveness of standard replacement therapy and often necessitates the use of bypassing agents, which are more expensive and less effective in preventing bleeding [14,15].

Multiple studies across different populations have reported on the frequency of the Inv22 mutation and its clinical relevance. For instance, its prevalence has been observed to be around 45% in European populations [6,7], 42%-50% in Middle Eastern countries [16], and 30%-40% in Asian cohorts, including India and China [17,18]. Furthermore, a systematic review by Gouw et al. (2012) highlighted that certain mutation types, particularly large deletions and Inv22, are strongly associated with a higher risk of inhibitor development [7].

Given the clinical implications of this genetic anomaly, particularly in relation to disease severity and treatment challenges, this study aims to investigate the frequency of the intron 22 inversion among patients with hemophilia A in a regional cohort. Additionally, it seeks to examine the association between this mutation and clinical parameters such as bleeding phenotype and inhibitor development.

## Materials and methods

This hospital-based observational study was conducted over a period of one year, from March 2023 to February 2024, with the objective of determining the frequency of intron 22 inversion among hemophilia A patients and assessing its correlation with disease severity. The study was carried out at Assam Medical College and Hospital (AMCH), Dibrugarh. Samples were collected from patients attending the Hemophilia Clinic of AMCH. Initial screening coagulation tests were performed in the Department of Pathology, and molecular testing, including detection of the intron 22 inversion mutation, was conducted in the Multi-Disciplinary Research Unit (MRU) under the Department of Microbiology, AMCH.

The study population included all diagnosed and newly diagnosed cases of hemophilia A attending the Hemophilia Clinic during the study period. Patients of all age groups, ranging from one year to 45 years of age, were included. The sample size was determined based on a previously reported prevalence of intron 22 inversion in hemophilia A patients, estimated at 23.3% [19]. Using a 95% confidence interval and an absolute margin of error of 10%, the calculated sample size was approximately 70.7. To account for any data loss or exclusions, this was rounded off to a final sample size of 80.

Inclusion criteria comprised all diagnosed and newly diagnosed hemophilia A patients attending the Hemophilia Clinic during the study period. Exclusion criteria included patients who did not provide informed consent and those diagnosed with hemophilia B or other inherited bleeding disorders. Ethical clearance for the study was obtained from the Institutional Ethics Committee (Human) of Assam Medical College and Hospital, Dibrugarh. Written informed consent was obtained from all participants or their legal guardians before enrollment.

Detailed clinical history and family history were obtained using a structured case proforma (see Appendix, Table 3), including demographic details, age at the first bleeding episode, frequency, severity, duration, joint or muscle bleeds, and history of inhibitor development. A thorough physical examination was performed to assess joint mobility and hemophilic arthropathy. Venous blood samples were collected from each patient under aseptic conditions in three tubes: an ethylenediamine tetraacetic acid (EDTA) tube (3 mL) for genomic DNA extraction and two citrated tubes (3.2% sodium citrate, 9:1 ratio) for coagulation tests and factor VIII activity measurement. EDTA samples were stored at 4°C for up to 48 hours before DNA extraction, while citrated plasma samples were centrifuged at 1700g for 15 minutes (15-22°C), separated, and stored at -80°C until analysis.

Coagulation assays were performed using the Erba Mannheim ECL 760 Coagulation Analyzer (Erba, Mannheim, Germany) for prothrombin time (PT) and activated partial thromboplastin time (APTT). Factor VIII activity was measured using a one-stage APTT-based assay on the Erba ECL 105 Semi-Automated Coagulation Analyzer (Erba Diagnostics, Mannheim, Germany), with normal pooled plasma (NPP) and factor VIII-deficient plasma as standards. Based on factor VIII activity, patients were categorized into severe (<1%), moderate (1-5%), and mild (5-40%) hemophilia A. Genomic DNA was extracted from whole blood (EDTA samples) using the QIAamp DNA Mini Kit (Qiagen, Hilden, Germany) following the manufacturer’s instructions, and DNA purity and concentration were assessed using a NanoDrop™ OneC Spectrophotometer (Thermo Fisher Scientific, Waltham, MA, USA).

To detect intron 22 inversion in the F8 gene, nested long-distance polymerase chain reaction (NLD-PCR) was performed using a T100 Thermal Cycler (Bio-Rad, Hercules, CA, USA), with the product of the first-round PCR serving as the template for the second-round nested PCR. Positive control samples known to carry the intron 22 inversion were included in each run to validate the specificity and sensitivity of the assay. In the first-round PCR, HemN-P, HemN-B, and HemN-Q primers were used with a reaction mix containing 10X buffer (2.2 µL), dNTP mix (0.8 µL), primers (0.4 µL each), expand-long DNA polymerase (Roche, Germany, 0.35 µL), template DNA (1 µL), and diethylpyrocarbonate (DEPC)-treated water (12.65 µL). 

Thermal cycling involves an initial denaturation at 94°C for one minute, followed by 10 cycles of 98°C for 10 seconds, 68°C for 12 minutes, then 20 cycles of 98°C for 10 seconds, 68°C for 12 minutes (+20 sec per cycle), and a final extension at 72°C for 10 minutes. The second-round nested PCR used HemN-P1, HemN-B1, and HemN-Q1 primers, with cycling conditions of 94°C for five minutes, followed by 30 cycles at 94°C (30 sec), 68°C (30 sec), 72°C (90 sec), and a final extension at 72°C for three minutes. PCR products were analyzed using 2% agarose gel electrophoresis on the Bio-Rad PowerPac™ Basic Electrophoresis System (Bio-Rad, Hercules, CA, USA), with a 100 bp DNA ladder as a molecular weight marker. A 540 bp band confirmed intron 22 inversion positivity [20].

## Results

Demographic profile and baseline characteristics

The study included 80 male patients diagnosed with hemophilia A, with a mean age of 17.06 ± 9.75 years (range: 2-39 years). The majority of cases (67.5%, n = 54) were below 20 years of age, with the highest proportion in the 0-10 years (n = 27, 33.75%) and 11-20 years (n = 27, 33.75%) groups, followed by 21-30 years (n = 16, 20.0%) and 31-40 years (n = 10, 12.5%).

A positive family history was noted in 52 cases (65.0%), while 28 patients (35.0%) had no known affected relatives, indicating the possibility of de novo mutations.

The most common clinical presentation was joint bleeding (hemarthrosis, n = 41, 51.25%), followed by intramuscular bleeding (n = 20, 25.0%), gum bleeding (n = 10, 12.5%), subcutaneous bleeding (n = 5, 6.25%), and epistaxis (n = 4, 5.0%). No cases of gastrointestinal, genitourinary, or central nervous system bleeding were recorded.

Screening coagulation tests showed a normal prothrombin time (PT) in all cases, while activated partial thromboplastin time (APTT) was significantly prolonged, with a mean APTT of 90.71 ± 20.36 sec (normal reference: 26-36 sec) (Table 1).

**Table 1:** Demographic and baseline characteristics of hemophilia A patients. Data presented as N (%) or mean ± SD. Statistical significance: p < 0.05 is considered significant. PT: prothrombin time, APTT: activated partial thromboplastin time.

Parameter	n (%)	Mean ± SD
Age group (years)		
0–10	27 (33.75)	
11–20	27 (33.75)	
21–30	16 (20.00)	
31–40	10 (12.50)	
Mean age		17.06 ± 9.75
Gender		
Male	80 (100.0)	
Female	0 (0.0)	
Family history		
Positive	52 (65.00)	
Negative	28 (35.00)	
Common clinical presentations		
Joint bleeding (hemarthrosis)	41 (51.25)	
Intramuscular bleeding	20 (25.00)	
Gum bleeding	10 (12.50)	
Subcutaneous bleeding	5 (6.25)	
Epistaxis	4 (5.00)	
Coagulation parameters		
PT (seconds)	Normal in all patients	
APTT (seconds)	Prolonged in all cases	90.71 ± 20.36

**Figure 1:**
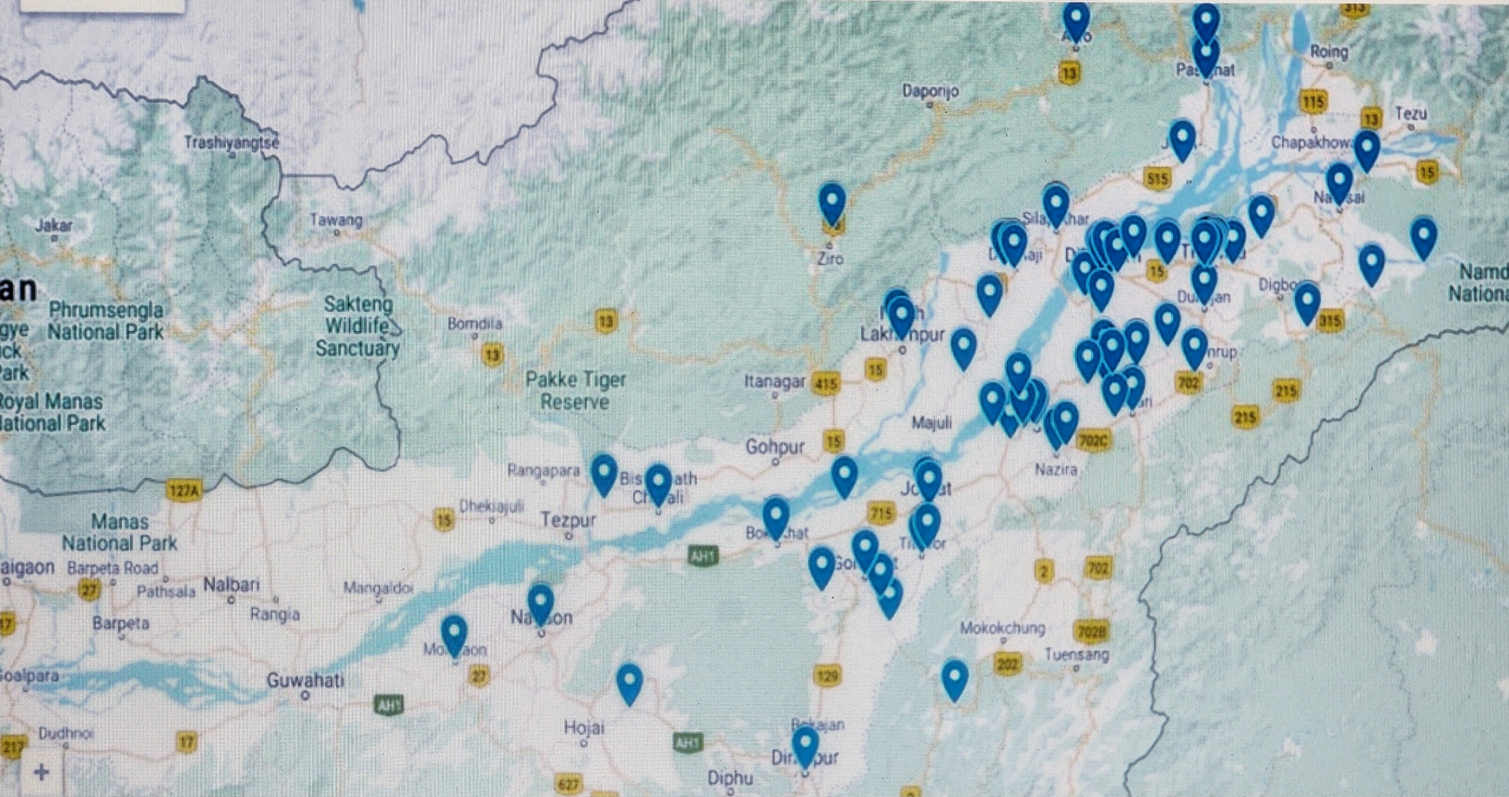
Location of hemophilia A patients in upper Assam region and nearby states of Northeast India. The image was created by authors' using Google Maps.

Most of our patients were from districts of upper Assam and nearby states of Northeast India (Figure 1).

Factor VIII activity was categorized as severe (<1%), moderate (1-5%), and mild (5-40%). Among the 80 patients, 41 (51.25%) had severe disease, 22 (27.5%) moderate, and 17 (21.25%) mild.

Nested long-distance PCR (NLD-PCR) revealed intron 22 inversion in 24 cases (30.0%). The mutation was present in 22 (53.65%) severe cases, 2 (9.09%) moderate cases, and zero mild cases. The chi-square test (χ² = 20.38, p < 0.001) confirmed a statistically significant association between intron 22 inversion and severe hemophilia A.

A comparison of APTT values across severity groups showed that severe cases had the longest mean APTT (109.35 ± 15.62 sec), followed by moderate (86.92 ± 9.87 sec) and mild (61.71 ± 8.94 sec) cases. A one-way ANOVA (F = 62.41, p < 0.001) confirmed a significant difference among the groups (Table 2).

**Table 2:** Factor VIII activity levels and Intron 22 inversion status in hemophilia A patients. Data represented as N (%) or Mean ± SD. Statistical significance: p < 0.05 is considered significant. APTT: activated partial thromboplastin time.

Severity group	n (%)	Intron 22 inversion positive (n, %)	APTT (mean ± SD, sec)	Test statistic (χ²/F-test)	p-value
Severe (<1% FVIII)	41 (51.25)	22 (53.65)	109.35 ± 15.62	χ² = 20.38	p < 0.001
Moderate (1–5% FVIII)	22 (27.50)	2 (9.09)	86.92 ± 9.87	F = 62.41	p < 0.001
Mild (5–40% FVIII)	17 (21.25)	0 (0.00)	61.71 ± 8.94		
Total	80 (100.0)	24 (30.0)	90.71 ± 20.36		

**Figure 2:**
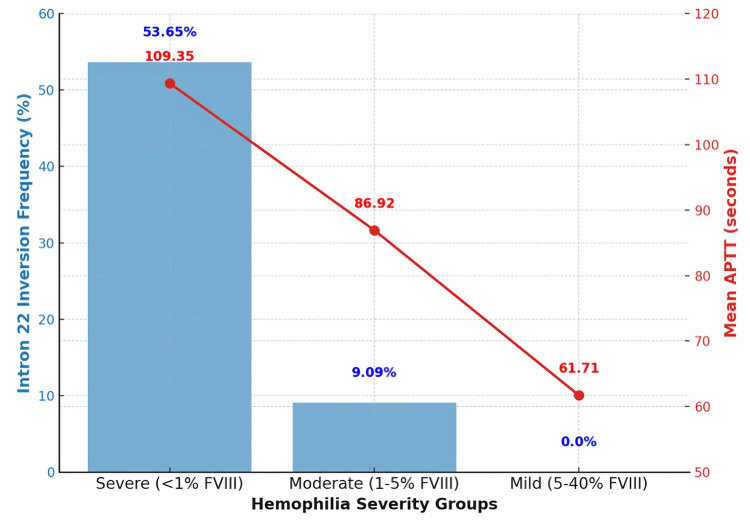
Intron 22 inversion frequency and APTT across severity groups. APTT: activated partial thromboplastin time.

The graphical representation illustrates the relationship between intron 22 inversion frequency and APTT values across hemophilia A severity groups. The blue bars indicate the percentage of cases with intron 22 inversion, showing a high prevalence (53.65%) in severe cases, a lower frequency (9.09%) in moderate cases, and a complete absence in mild cases. The red line with markers represents the mean APTT values, which increase progressively with disease severity (109.35 sec in severe, 86.92 sec in moderate, and 61.71 sec in mild cases). Figure 2 highlights the strong correlation between intron 22 inversion and severe hemophilia A, reinforcing its role in disease progression and severity classification.

## Discussion

The present study investigates the prevalence of intron 22 inversion in hemophilia A patients in Assam, India, and provides a comparative analysis with similar studies conducted in India and globally. Hemophilia A is the most common inherited bleeding disorder, caused by mutations in the F8 gene, with intron 22 inversion accounting for nearly 45% of severe cases worldwide [6]. Our study found a significant correlation between intron 22 inversion and disease severity, aligning with global reports [7].

In our study, 80 hemophilia A patients were assessed, with 51.25% presenting with hemarthrosis, followed by muscle hematomas (25%) and gum bleeding (12.5%). A study in India by David et al. found hemarthrosis in 48% of patients, closely matching our findings [21]. Similarly, a study in North India by Phadke reported hemarthrosis as the most common presentation (52%), confirming the trend of joint involvement as a major clinical feature in Indian hemophilia patients [22].

Globally, studies have demonstrated a variable prevalence of intron 22 inversion. In a European cohort, Antonarakis et al. reported a 43% prevalence of intron 22 inversion in severe hemophilia A cases [23]. In a Chinese study, Lyu et al. identified a 40.2% prevalence, while a South African study by Kloppers et al. documented a 47% prevalence [24]. Our study found a slightly lower prevalence, which may be attributed to regional genetic variations and the sample size. However, our results are in concordance with studies conducted in the Indian subcontinent [25].

The detection of intron 22 inversion using nested long-distance PCR was done in our cohort. A similar methodological approach was used by Lyu et al. in a targeted sequencing study, demonstrating the robustness of this technique [25]. The specificity and sensitivity of this method make it an essential tool in genetic diagnostics for hemophilia A. Additionally, our findings on factor VIII activity levels correspond well with previous research showing that severe cases (factor VIII <1%) have the highest likelihood of carrying intron 22 inversion [26,27].

The presence of family history in 65% of our cases is consistent with previous studies from India and other developing countries. Studies from the Middle East and North Africa region report familial clustering in over 70% of cases, indicating similar genetic inheritance patterns across populations [1]. This suggests that genetic counseling should be a crucial aspect of hemophilia care in resource-limited settings, as emphasized by the World Federation of Hemophilia [28].

Our study also highlights the importance of early diagnosis and comprehensive care. Given the high prevalence of intron 22 inversion among severe hemophilia A patients, molecular testing should be integrated into routine diagnostic workflows, as suggested by Gouw et al. [29]. The increasing availability of molecular diagnostics in India presents an opportunity to improve hemophilia management through early genetic screening and targeted therapeutic interventions.

Strengths and limitations of the study

A major strength of this study lies in its focus on a relatively underrepresented population in Northeast India, thereby contributing novel and region-specific data to the understanding of hemophilia A genetics. The use of nested long-distance PCR (NLD-PCR) offered high diagnostic sensitivity and specificity for detecting intron 22 inversion, enhancing the reliability of the genetic results. Additionally, comparison with national and international datasets helped contextualize the observed prevalence and provided meaningful insights into regional variation.

The study's sample size of 80 patients was calculated based on a previously reported intron 22 inversion prevalence of 23.3%, with a 95% confidence interval and a 10% absolute margin of error, making it statistically adequate to estimate prevalence. However, the sample size may not have been sufficient to detect subtle or subgroup differences, such as genotype-phenotype correlations or inhibitor development patterns, thus limiting the statistical power for more granular analyses.

Potential confounding factors, such as consanguinity, genetic drift, or environmental influences, were not assessed in this study but could play a role in the observed mutation prevalence and should be explored in future investigations. Furthermore, the study did not employ functional assays or RNA-based techniques to directly assess the impact of intron 22 inversion on Factor VIII gene expression or mRNA stability. Incorporating such functional validations in future studies, including RNA sequencing or quantitative transcript analysis, would provide deeper insights into the biological consequences of this mutation.

Lastly, while the current study focused on the molecular detection of intron 22 inversion, it did not evaluate the presence of inhibitors or the clinical response to factor replacement therapy. Given the established association between certain F8 gene mutations and inhibitor development, future research should integrate immunological profiling and treatment outcome data to better understand the clinical implications of intron 22 inversion and to guide personalized therapeutic strategies.

Future scopes of research

Future research should aim to expand the genetic screening of hemophilia A patients by incorporating advanced molecular techniques, such as targeted next-generation sequencing (NGS) panels specific to the F8 gene and its regulatory regions. Broader approaches, including whole-exome or whole-genome sequencing, may also be considered to uncover rare or novel mutations that contribute to disease severity, especially in patients who test negative for common mutations like intron 22 inversion.

A larger, multicentric study encompassing diverse ethnic populations across India would provide a more comprehensive understanding of regional and genetic heterogeneity in mutation prevalence and disease expression. Such efforts could also help identify potential population-specific founder mutations or modifier genes.

Additionally, the influence of non-genetic factors, including environmental exposures, nutritional status, and potential epigenetic modifications, should be explored as they may modulate the clinical phenotype and variability in bleeding severity among patients with similar genotypes. Investigating the role of DNA methylation, histone modifications, and non-coding RNAs could offer new insights into gene regulation in hemophilia A.

Further research should also focus on evaluating the impact of specific mutations, such as intron 22 inversion, on treatment response and inhibitor development, facilitating the implementation of individualized therapy protocols. The integration of functional studies such as RNA sequencing or proteomic analysis can provide a better understanding of how mutations affect FVIII expression at the transcript and protein levels.

Emerging therapeutic strategies warrant exploration in future studies. These include CRISPR-Cas9-based genome editing for correcting F8 mutations at the DNA level, mRNA-based therapies to provide transient expression of functional FVIII, and the development of bispecific antibodies (e.g., emicizumab) that can mimic FVIII cofactor activity. Such innovations hold the promise of transforming the management of hemophilia A, particularly for patients with inhibitor complications or those who are not candidates for conventional factor replacement therapy.

## Conclusions

This study is one of the first to investigate the prevalence of intron 22 inversion among hemophilia A patients in Assam, Northeast India, highlighting a significant genetic contributor to severe disease in this region. The findings, consistent with national and global data, emphasize the critical role of early molecular diagnostics in guiding genetic counseling and individualized management. By demonstrating a 23.75% prevalence of intron 22 inversion and its strong association with severe phenotypes, the study underscores the need to integrate targeted genetic screening, such as nested long-distance PCR or targeted NGS panels, into routine clinical protocols for hemophilia A care.

Healthcare facilities, especially in resource-limited settings, should prioritize the establishment of regional molecular diagnostic centers and train personnel to support early identification and counseling. Furthermore, the absence of intron 22 inversion in many mild and moderate cases calls for future exploration of additional genetic variations, epigenetic modifiers, or environmental influences that may affect disease severity. A broader, multi-pronged approach will deepen our understanding of the clinical heterogeneity of hemophilia A and ultimately improve patient outcomes across the spectrum.

## Appendices

**Table 3:** Case proforma.

Case No
Hosp No:
1. MRD No:
2. Ward/OPD:
Name:
1. Age:
2. Sex:
Ethnicity:
Date of examination:
Hemophilia A diagnosed on:
Disease duration:
Present complaints:
Bleeding events in past:
Family history:
Systemic:
1. Fever:
2. Fatigue:
3. Anorexia:
4. Weight loss:
5. Palpable lymph nodes (cervical/inguinal/axillary):
Systemic examination:
1. Musculoskeletal
2. Mucocutaneous involvement
3. Gastrointestinal
4. Genitourinary
5. Nervous system
6. Cardiovascular system
7. Respiratory system
Provisional/clinical diagnosis:
Laboratory examination:
1. Hematological:
Complete blood count:
Coagulation parameters
Prothrombin time
Activated plasma thromboplastin time
Factor VIII activity
(i) FVIII <1%
(ii) FVIII 1-5%
(iii) FVIII 5-<40%
2. Molecular interpretation (Intron 22 inversion)
